# Genome‐scale metabolic modelling of SARS‐CoV‐2 in cancer cells reveals an increased shift to glycolytic energy production

**DOI:** 10.1002/1873-3468.14180

**Published:** 2021-09-05

**Authors:** Elisabeth Yaneske, Guido Zampieri, Loris Bertoldi, Giuseppe Benvenuto, Claudio Angione

**Affiliations:** ^1^ School of Computing, Engineering and Digital Technologies Teesside University Middlesbrough UK; ^2^ Department of Biology University of Padua Italy; ^3^ BMR Genomics Padua Italy; ^4^ Healthcare Innovation Centre Teesside University Middlesbrough UK; ^5^ Centre for Digital Innovation Teesside University Middlesbrough UK

**Keywords:** cancer, COVID‐19, flux balance analysis, genome‐scale metabolic modelling, multi‐omics, SARS‐CoV‐2

## Abstract

Cancer is considered a high‐risk condition for severe illness resulting from COVID‐19. The interaction between severe acute respiratory syndrome coronavirus‐2 (SARS‐CoV‐2) and human metabolism is key to elucidating the risk posed by COVID‐19 for cancer patients and identifying effective treatments, yet it is largely uncharacterised on a mechanistic level. We present a genome‐scale map of short‐term metabolic alterations triggered by SARS‐CoV‐2 infection of cancer cells. Through transcriptomic‐ and proteomic‐informed genome‐scale metabolic modelling, we characterise the role of RNA and fatty acid biosynthesis in conjunction with a rewiring in energy production pathways and enhanced cytokine secretion. These findings link together complementary aspects of viral invasion of cancer cells, while providing mechanistic insights that can inform the development of treatment strategies.

## Graphical abstract

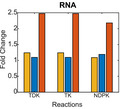

## Abbreviations

**AGP**, alpha‐1 acid glycoprotein

**COVID‐19**, coronavirus disease 2019

**DARs**, differentially active reactions

**DEGs**, differentially expressed genes

**DEPs**, differentially expressed proteins

**dUMP**, deoxyuridine monophosphate

**FBA**, flux balance analysis

**FVA**, flux variability analysis

**GO**, gene ontology

**GSMMs**, genome‐scale metabolic models

**hpi**, hours post‐infection

**IP10**, interferon γ‐induced protein‐10

**MIP‐1β**, macrophage inflammatory protein‐1β

**PPARalpha**, peroxisome proliferator‐activated receptor alpha

**SARS‐CoV‐2**, severe acute respiratory syndrome coronavirus‐2

**SDH**, succinate dehydrogenase

**VEGFA**, vascular endothelial growth factor A

The rapid spread of severe acute respiratory syndrome coronavirus‐2 (SARS‐CoV‐2) has created a global public health emergency that is currently affecting countries across the globe [1, 2]. The coronavirus disease 2019 (COVID‐19) pandemic poses challenges to healthcare systems due to the lack of specific therapeutics for prevention or patient treatment. In turn, drug development requires scientists across biomedical disciplines to expand our knowledge on biomolecular mechanisms regulating SARS‐CoV‐2 infection and the associated cascade of cellular alterations.

Cancer is considered by the Centers for Disease Control and Prevention a high‐risk condition for severe illness resulting from COVID‐19. However, it is not clear to what extent this comorbidity affects the cellular mechanisms observed in both diseases. While cancer patients infected with SARS‐CoV‐2 are known to be at increased risk, the human metabolic response to SARS‐CoV‐2 infection in cancer cells remains largely uncharacterised to date [3, 4, 5, 6]. The severity of physiological symptoms experienced by individuals is largely dependent on the host response, which means understanding the metabolic changes caused by infection is crucial to identifying treatments for managing both the immediate and longer‐term effects on health [7].

Further, liver dysfunction has been reported to increase with increasing severity of COVID‐19 infection [8, 9, 10, 11, 12]. It has been reported that the ACE2 receptor that facilitates viral entry to the cell is highly expressed in cholangiocytes and is also expressed in hepatocytes to a lesser extent [13, 14]. Chu *et al*. [15] demonstrated that COVID‐19 can replicate within liver cancer cells such as Huh7. However, the precise mechanism underlying the viral effect on the liver has still to be determined [16, 17].

Recently, time‐course transcriptomic measurements of SARS‐CoV‐2‐infected Huh7 cells have been reported [18]. In this study, the ErbB, HIF‐1, mTOR and TNF signalling pathways were identified as significantly modulated during the course of the SARS‐CoV‐2 infection. The mTOR signalling pathway was thus suggested as a potential drug target to treat COVID‐19 patients. mTOR is known to be implicated in the metabolic control of glucose, nucleotides and lipids [19]. This study thus highlights the relevance of omics analyses for the consolidation of knowledge with potential for clinical translation. Studies based on gene and protein transcription levels, however, provide scattered information that may be difficult to unify and interpret.

Similarly, studies of circulating blood metabolites in COVID‐19 patients have yielded interesting biomarkers of infection, including hijacking of nucleic acid intermediates [20, 21, 22, 23], dysregulation of lipid metabolism [22, 24, 25], changes in amino acid metabolism [23, 25], alteration of energy metabolism [26], immune response [27], and indicators of hepatic cell damage [25]. However, a limitation of metabolomics alone is that it focuses on alterations of metabolites at a pathway level rather than identifying altered reaction/enzyme activity, which allows for more specific therapeutic targeting.

To unify and interpret heterogeneous omics data, systems biology offers powerful tools such as genome‐scale metabolic modelling [28]. This approach mathematically describes metabolic networks and their activity by unifying large corpuses of detailed biochemical knowledge, thus allowing the estimation of metabolic phenotypes on a single‐reaction resolution [29]. For instance, previous studies exploited it for predicting biomarkers in the context of cancer and other diseases [30, 31, 32]. In the context of SARS‐CoV‐2, there have been few studies to investigate the metabolic effects of viral infection at genome scale [33, 34] and to our knowledge, none that investigate the metabolic effects of infection in cancer cells, an aspect of special relevance in cancer [35].

**Fig. 1:**
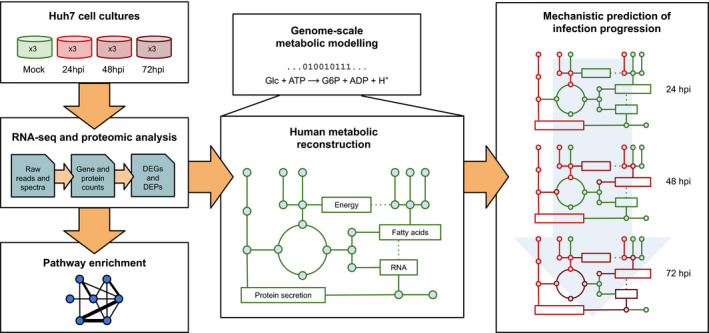
Workflow followed in the study. Time series RNA‐seq and proteomic data from infected Huh7 cells sampled at 24, 48 and 72 hours post‐infection (hpi) were analysed and used to identify DEGs, DEPs and dysregulated pathways compared with uninfected cultures. Results from RNA‐seq and proteomic analysis were used to inform a genome‐scale metabolic reconstruction of human metabolism, which was expanded to include translation and secretion pathways for a range of immune proteins alongside a SARS‐CoV‐2 biomass objective function. Gene and protein expression counts were thus incorporated in the baseline model to capture time‐dependent changes in metabolic activity by estimating DARs with respect to uninfected Huh7 cells.

To address these gaps, we apply a systems biology approach to explore how SARS‐CoV‐2 infection impacts human cancer cell metabolism and how distinct pathways are affected over time. To this end, we build genome‐scale metabolic models (GSMMs) that incorporate multiple transcriptional and protein expression states after infection and that reflect consecutive stages of immune response to viral invasion. Thanks to a flux balance analysis (FBA) framework, such models allowed us to explore in detail how the disease insurgence impacts single metabolic reactions. Through this approach, we identify altered pathway capabilities and reconstruct a global overview of metabolic rewiring under SARS‐CoV‐2 infection (Fig. 1 for the workflow).

Our analysis identified four main areas of metabolism affected by SARS‐CoV‐2 infection: RNA production, energy production, fatty acid metabolism and the secretome. Glycolysis showed upregulated production of ATP while the mitochondrial electron transport chain and oxidative phosphorylation were downregulated. This diversion of energy production through glycolysis is in addition to the upregulation of glycolysis already known to occur in cancer cells. The amplified effect of cancer metabolism may explain why cancer patients are at higher risk from SARS‐CoV‐2 infection. Synthesis of fatty acids for the viral cell envelope was increased, while unsaturated fatty acid synthesis was decreased. Secretion of interferon γ‐induced protein‐10 (IP10/CXCL10), which is associated with the cytokine storm, was highly upregulated. Finally, we suggest some biological processes that could be targeted by therapeutic treatments based on our findings.

## Materials and methods

### Data collection

RNA‐seq data were taken from the SRA database (accession PRJNA627100). Briefly, Huh7 cells were used to perform transcriptomic analysis in uninfected and 24, 48 and 72 hours post‐infection (hpi) by the SARS‐CoV‐2 virus. Three specimens for each time point were collected. Complete data production is extensively reported in the original paper of Appelberg *et al*. [18]. Preprocessed proteomic data for Huh7 cells at the same time points were obtained from the authors.

### RNA‐seq and proteomic data processing and analysis

RNA read preprocessing was performed by using fastp v0.20.0 [36], applying specific parameters in order to remove residual adapter sequences and to keep only high‐quality data (qualified_quality_phred = 20, unqualified_percent_limit = 30, average_qual = 25, low_complexity_filter = True, complexity_threshold = 30). Then, passing filter reads were mapped to the human genome reference (version GRCh38) using star v2.7.0 [37] with standard parameters, except for sjdbOverhang option set to 74 due to read length. Genome and transcript annotation provided as input were downloaded from the Ensembl repository v99 (genome [38], annotation [39]). Alignments were then elaborated by rsem v1.3.3 [40] to estimate transcript and gene abundances. Subsequently, sample‐specific gene‐level abundances were merged into a single raw expression matrix applying a dedicated rsem command (rsem‐generate‐data‐matrix). Genes with less than 10 counts in all samples were then filtered out. Gene differential expression (pairwise comparisons) was computed by edger [41] from raw counts in each comparison, following the authors' instructions. A multiple testing correction was applied (estimateDisp, glmQLFit and glmQLFtest), and genes with a *P*‐value ≤ 0.05 were considered differentially expressed.

Starting from quantile‐normalised protein abundance, proteomic profiles were processed as described in the original paper [18]. Proteomic‐transformed data were processed through linear models using the limma r package [42]. In the limma design matrix, separated coefficients were associated with time and samples in order to define the contrasts. For the pairwise comparisons, a single factorial design was implemented to fit models with a coefficient for each of four factors: uninfected, 24, 48 and 72 hpi. As a result, significant differentially expressed proteins were selected based on *P*‐values after a Benjamini–Hochberg (BH) adjustment. Proteins with an alpha value less than 0.05 were considered significant. For both proteomic and transcriptomic results, no threshold was applied on the log2 fold change.

Re‐annotation of differentially expressed genes (DEGs) and proteins was performed using the biomart package [43] in r 3.6, querying available Ensembl Gene IDs and retrieving Gene Names and HGNC gene IDs to allow mapping of genes onto the metabolic model. Then, GO (gene ontology) and pathway enrichment analyses were performed on the KEGG [44] and Reactome [45] public pathway databases, which consist of graphical diagrams of biochemical pathways including most of the known metabolic reactions.

### Expansion of a human genome‐scale metabolic reconstruction

We modelled the hepatocyte‐derived cellular carcinoma cell line Huh7 metabolism based on the Recon 2.2 genome‐scale reconstruction [46]. This model incorporates a large number of biochemical pathways of human metabolism and is the result of joint efforts by many scientists and several rounds of curation. We expanded Recon by including translation and secretion pathways for relevant proteins using a recently introduced pipeline [47]. Secretory proteins were selected based on the secretomes of COVID‐19 [11, 12, 48, 49, 50], SARS [51, 52, 53], oxidative stress [54] and liver cells [55] (Table 1). Moreover, we introduced a SARS‐CoV‐2 biomass pseudoreaction accounting for viral metabolic requirements within host cells, to enable an integrated human virus metabolic modelling simulation [56]. Specifically, we employed a biomass stoichiometry defined in previous works by assembling molecular investments in SARS‐CoV‐2 biomass and that accounts for nucleotide, amino acid, lipid and energetic requirements [33, 34]. The resulting model spans 1838 genes, 9892 reactions and exchange pseudoreactions and 7386 metabolites across 10 cellular compartments.

**Table 1:** Genome‐scale metabolic model secretory proteins. Table showing the name, gene and protein UniProt ID for the secretome added to the GSMM.

Name	Gene	Protein
Alpha‐1 acid glycoprotein	*ORM1*	P02763
Apolipoprotein A1	*APOA1*	P02647
Basic fibroblast growth factor	*FGF2*	P09038
Chemokine (C‐C motif) ligand 20	*CCL20*	P78556
C‐reactive protein	*CRP*	P02741
Eotaxin	*CCL11*	P51671
Fetuin	*AHSG*	P02765
Granulocyte colony‐stimulating factor (GSCF)	*CSF3*	P09919
Haptoglobin	*HP*	P00738
IFN‐γ	*IFNG*	P01579
Proinflammatory interleukins IL‐1β, IL‐2, IL‐5, IL‐6, IL‐8, IL‐12A, IL‐12B, IL‐15, IL‐17A	*IL1B, IL‐2, IL‐5, IL‐6, CXCL8, IL‐12A, IL‐12B, IL‐15, IL‐17RA*	P01584, P60568, P05113, P10145, P29459, P29460, P40933, Q96F46
Anti‐inflammatory interleukins IL1RA, IL‐4, IL‐7, IL‐9, IL‐10, IL‐13	*IL1RN, IL‐4, IL‐7, IL‐10, IL‐13, IL‐9*	P18510, P05112, P05231, P13232, P22301, P35225, P15248
Interferon γ‐induced protein‐10 (IP10)	*CXCL10*	P02778
Macrophage inflammatory protein‐1α (MIP‐1α)	*CCL3*	P10147
Macrophage inflammatory protein‐1β (MIP‐1β)	*CCL4*	P13236
Monocyte chemoattractant protein‐1 (MCP1)	*CCL2*	P13500
Peroxiredoxin‐1	*PRDX1*	Q06830
Peroxiredoxin‐2	*PRDX2*	P32119
Platelet‐derived growth factor subunit B	*PDGFB*	P01127
RANTES	*CCL5*	Q9Y2Y9
Thymic stromal lymphopoietin	*TSLP*	Q969D9
Transferrin	*TF*	P02787
Transthyretin	*TTR*	P02766
Tumour necrosis factor‐α (TNF‐α)	*TNF*	P01375
Vascular endothelial growth factor A (VEGFA)	*VEGFA*	P15692
α1‐Antichymotrypsin	*SERPINA3*	P01011
α1‐Antitrypsin (TF‐α1‐AT)	*SERPINA1*	P01009

### Infection‐stage‐specific metabolic modelling

Flux balance analysis is a mathematical approach for analysing the flux of biochemical reactions through a genome‐scale reconstruction of metabolic networks [28, 29]. FBA requires a genome‐scale metabolic network reconstruction, represented as a stoichiometric matrix **S** where the rows correspond to metabolites and the columns represent reactions. Under a steady‐state assumption, there is no net change in mass in the system and the mass is conserved. Therefore, the rate of production of each internal metabolite equals its rate of consumption. A column vector ***v*** represents the flux through the system (reaction rate of each reaction). Under the steady‐state assumption, the matrix multiplication of the stoichiometric matrix **S** and column vector ***v*** provides the linear equations representing the constraints (**S*v*** = 0).

Further constraints are added through RNA‐seq expression profiles, and specifically the lower and upper bounds of each metabolic flux lb ≤ ***v*** ≤ ub. These are vectors representing the lowest and highest reaction rate possible for each reaction. These constraints reduce the possible solution space and can be set for each condition, therefore creating condition‐specific metabolic models [29].

Temporal progression was modelled through the creation of condition‐specific models, based on the gene and protein expression fold change profiles over the three time points. Gene expression for the unaffected cells was used as a control when calculating the fold changes. Condition‐specific models were generated through a modified version of metrade [57] where flux bounds are a linear function of gene set expression values. This process requires, as input, expression fold changes, which are then converted into reaction flux bounds through gene‐protein‐reaction rules encoded in the model. The same process was repeated with proteomic data, therefore generating two sets of models, which will be denoted by transcriptomic‐informed and proteomic‐informed GSMMs. The flux bounds obtained after solving these models were then used in the simulations described below.

### Simulating alterations in metabolic network capabilities

In addition to the transcriptional constraints described above, we utilised additional constraints devised based on the literature and our RNA‐seq analysis results. Regulation of lipid metabolism by peroxisome proliferator‐activated receptor alpha (PPARalpha), which increases the size and number of peroxisomes, was identified from the pathway analysis. PPARalpha is known to be involved in regulating fatty acid metabolism and the immune response in hepatocytes [58]. Acyl‐CoA oxidase is a rate‐limiting enzyme activated by PPARalpha [59]. Two further constraints were added from a review of the literature and their correlated increase in activity with the viral infection. Phosphogluconate dehydrogenase catalyses the conversion of 6‐phosphogluconate to ribulose‐5‐phosphate in the cytosol generating NADPH. Ribulose‐5‐phosphate is used for nucleotide biosynthesis. The reaction catalysed by phosphogluconate dehydrogenase is increased when there is a need for nucleotide and fatty acid synthesis, which uses the NADPH, such as for viral replication [60, 61]. The third and final reaction chosen occurs in peroxisomes, where lactate and NAD+ are converted to pyruvate and NADH by NAD+ oxidoreductase. The pyruvate/lactate ratio is linked with the NAD+/NADH ratio [62], potentially suggesting that the pyruvate is then shuttled back into the cytosol to be recycled into the citric acid cycle. Following the evidence described above, we set the lower bound of phosphogluconate dehydrogenase, acyl‐CoA oxidase and NAD+ oxidoreductase to 80% of their maximum value. Moreover, to account for cells growing in a culture, we constrained biomass production to be at least 50% of its maximum value. Analogously, we set a lower bound on viral biomass production to simulate the effects of intracellular viral replication, based on a total virion dry weight estimate of one‐third the host cell's at peak infection [63]. The viral biomass production was therefore constrained to be above a variable fraction of the maximum host biomass across the time points, in order to get a peak bound of one‐third of its value for the 72‐h time point. Only for the translation and secretion pathways, we used the same bound over all the time points, in order to best balance the impact of the viral biomass. Finally, oxygen uptake was set as unlimited, while oxygen production and superoxide consumption were blocked.

After having obtained a constrained model, flux variability analysis (FVA) was used to evaluate the minimum and maximum value of the flux that each reaction can carry, while still satisfying the given constraints. FVA was run by solving a double linear programming problem on each condition‐specific metabolic model. Estimation of metabolic potential on each time point was performed by FVA as implemented in the COBRA toolbox [64]. A metabolic network visualisation of flux fold changes was generated using ReconMap [65].

### Statistical analysis of differentially active reactions

For each time point, we determined the most perturbed metabolic pathways by a one‐sided hypergeometric test with false discovery rate correction for multiple testing on the differentially active reactions (DARs). Overactive DARs were defined as those reactions whose maximal flux fold change was 1.5 or greater and above the 95th percentile of the fold change distribution. For underactivity, we considered reactions whose maximal flux fold change was 0.8 or lower and fell in the 5th fold change percentile. In this way, we could select metabolic reactions characterised by activity changes with both statistical relevance and meaningful effect size.

## Results and Discussion

### Reconstructing the metabolic evolution of SARS‐CoV‐2 infection

To evaluate how SARS‐CoV‐2 infection affects metabolic activity along short‐term post‐infection time points (0, 24, 48 and 72 h), we applied our condition‐specific modelling approach, as described in the Materials and methods section and illustrated in Fig. 1. Integrated with machine learning methods, such an approach has previously revealed insights in infectious and complex disorders [66, 67, 68, 69]. Our modelling and analyses steps were performed starting from the multi‐omics dataset recently published by Appelberg *et al*. [18].

**Fig. 2:**
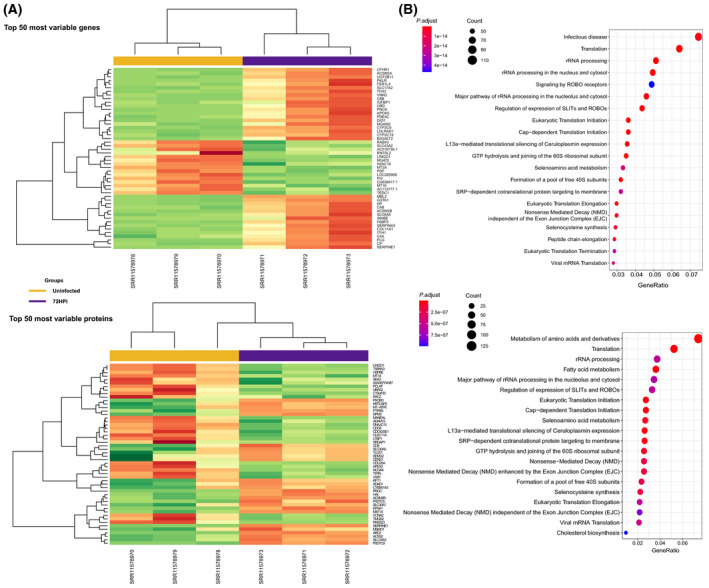
Dysregulated DEGs and DEPs identified by RNA‐seq and proteomic analysis. (A) Hierarchical clustering heatmap representing the most variable genes (|logFC| > 2) considering their transcript (top) and protein (bottom) expression in uninfected (yellow) and 72 hpi (violet) samples. Overexpression is red coloured, while underexpression is depicted in green. (B) Enrichment analysis showing the most relevant KEGG and Reactome pathways populated by differentially expressed genes (top) and proteins (bottom). Circle size indicates the number of genes involved in each pathway. Colour gradient represents statistical significance based on adjusted *P*‐value.

As a result of transcriptomic analysis, we obtained a high percentage of uniquely mapped reads, ranging from 88.02 and 89.13, with a mean value of 88.5. Then, we calculated DEGs among all the possible time point combinations, focusing on the differences between uninfected cells and the most advanced stage of virus incubation (72 hpi), which is expected to disclose the most valuable and significant results. Analogously, we obtained DEPs for the same contrasts. At 72 hpi, we identified 3464 DEGs and 883 DEPs (Table S1), associated with a large number of biological pathways (Fig. 2), including upregulated glycolysis and inflammatory secretome as found previously [18]. In addition, we identified dysregulation in the biosynthesis of amino acids and fatty acid metabolism, biomarkers of hepatic cell injury and biomarkers of endoplasmic reticulum stress due to increased demand for protein folding [70]. The enrichment results are listed in Tables S2 and S3. DEGs and DEPs derived from the comparisons across time points were used to inform the genome‐scale model in order to specifically highlight metabolic alterations due to the SARS‐CoV‐2 infection.

To obtain a mechanistic understanding of the alterations observed on a transcriptional level, we integrated the gene and protein expression profiles described above within Recon 2.2, a GSMM of human cells. The selected GSMM is among the most complete templates for the human metabolic network and was chosen based on its manually curated pathways and the validated representation of energy generation across various carbon sources [46]. As Recon 2.2 is a general‐purpose GSMM, it incorporates all the main metabolic pathways for a range of tissues and can be tailored to any target tissue through integration with omics data, as reviewed elsewhere [29, 71, 72]. Moreover, we expanded the GSMM by including a SARS‐CoV‐2 biomass accumulation pseudoreaction and secretory pathways for a range of relevant immune proteins (see Materials and methods). Our multi‐omics genome‐scale metabolic modelling approach thus allows us to investigate key dysregulated enzymes and reactions, giving a more holistic view of the metabolic phenotype of the infected cancer cell.

**Fig. 3:**
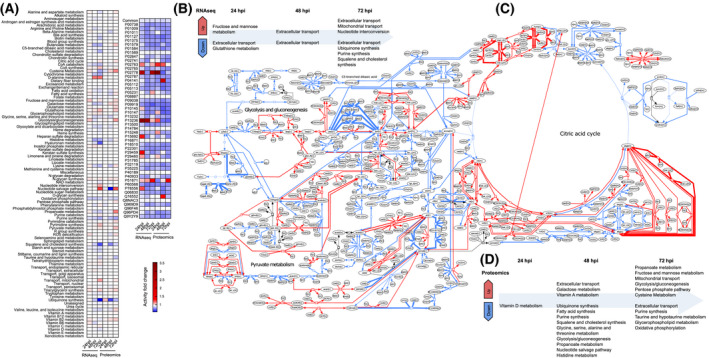
Genome‐scale metabolic modelling predicts alterations in multiple pathways following SARS‐CoV‐2 infection. (A) Pathway‐level temporal progression of Huh7 cell metabolic capabilities up to 72 h from infection. (B) Pathway enrichment of DARs identified at each time point in transcriptomic‐informed GSMMs. (C) Visualisation of alterations in central metabolism after 72 h from infection. (D) Pathway enrichment of DARs identified at each time point in proteomic‐informed GSMMs. In all the panels, red denotes increased activity potential for biochemical reactions and pathways, while blue indicates reduced activity.

Our modelling approach assumes a metabolic steady state, which can be assumed by considering different stages of infection that are distant in time. This approach has previously been used to detect biomarkers and drug targets for a range of disorders [30, 31, 73]. The result is a global picture of metabolic capabilities associated with varying transcriptional activity at each time point (24, 48 and 72 h). Uninfected cells were used as a control to analyse the changes in metabolic capabilities (see Materials and methods). Figure 3A shows the average activity fold change across reactions for all the pathways in the human metabolic network, while detailed results can be found in Tables S4 and S5. Viral metabolic perturbations affect a range of pathways including amino acids, energy production and coenzymes, which appear directly linked to specific protein oversecretion. In Fig. 3C, we visualise the activity of main energy production pathways, providing a graphical account of local alterations. Further, Fig. 3B,D shows pathways enriched in DARs at each time point, respectively, based on RNA‐seq and proteomic data integration.

To obtain a global overview of how SARS‐CoV‐2 affects cancer cell metabolism, we closely inspected altered pathways and individual reactions. In the following sections, we describe the main patterns identified and discuss their potential role in SARS‐CoV‐2 invasion inside host cells.

### Genome‐scale modelling identifies RNA production upregulation

**Fig. 4:**
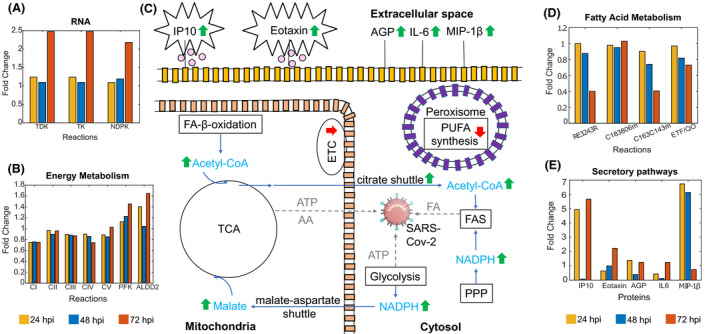
Summary of main metabolic changes in SARS‐CoV‐2‐infected Huh7 cells at 72 hpi. The mitochondrial ETC and oxidative phosphorylation are downregulated, reducing ATP production from the TCA cycle but potentially providing protection from apoptosis. The components of the TCA producing intermediates for viral amino acid synthesis are upregulated. While the production of ATP is reduced in the TCA, the energy‐producing reactions of glycolysis are upregulated, producing ATP for the viral production of amino acids and RNA. Fatty acid synthesis is also upregulated, producing fatty acids for the synthesis of the viral envelope. The production of anti‐inflammatory polyunsaturated fatty acids and the electron transport chain are both downregulated. Upregulation of inflammatory proteins IP10, eotaxin, MIP‐1β and IL‐6 has been linked with the cytokine storm. AGP is an acute‐phase protein involved in the inflammatory response. Green upward arrows represent upregulation. Red downward arrows represent downregulation. (A) Upregulated transcriptomic‐informed flux reactions involved in RNA production. (B) Dysregulated transcriptomic‐informed flux reactions involved in oxidative phosphorylation and glycolysis. (C) Visual overview of main metabolic changes. (D) Dysregulated transcriptomic‐informed flux reactions involved in fatty acid metabolism; RE3243R, production of polyunsaturated fatty acids, for example oleic acid; C183806m, final step of β‐oxidation producing octanyl‐CoA; C163C143m, first step of β‐oxidation; ETF/QO, electron transfer from octanoyl‐CoA to ubiquinone. (E) Upregulated transcriptomic‐informed secretory pathways involved in the immune response. PPP, pentose phosphate pathway; FAS, fatty acid synthesis; FA, fatty acid, TCA, citric acid cycle; AA, amino acid; FA‐β‐oxidation, fatty acid‐β‐oxidation; ETC, electron transport chain; PUFA, polyunsaturated fatty acid; IP10, interferon γ‐induced protein‐10; AGP, alpha‐1 acid glycoprotein; IL‐6, interleukin 6; MIP‐1β, macrophage inflammatory protein‐1β; TDK, deoxyuridine kinase; TK, thymidine kinase; NDPK, NDP kinase; C183806m, FAOXC183806m; C163C143m, FAOXC163C143m; ETF/QO, electron transfer flavoprotein/electron transfer flavoprotein–ubiquinone oxidoreductase, CI, NADH: ubiquinone oxidoreductase; CII, succinate dehydrogenase; CIII, cytochrome reductase; CIV, cytochrome c oxidase; CV, ATP synthase; PFK, phosphofructokinase; ALDD2, aldehyde dehydrogenase.

The nucleotide interconversion pathway shows evidence of increasing the uracil available to produce RNA by ensuring that the dUTP/dTTP ratio is low to minimise uracil incorporation into DNA [74]. The two top upregulated reactions at 72 hpi in the transcriptomic‐informed flux data are the catalysis of uridine monophosphate (UMP) to deoxyuridine monophosphate (dUMP) by deoxyuridine kinase and of thymidine to dTMP (deoxythymidine monophosphate) by thymidine kinase (see TDK and TK in Fig. 4A). dUMP is a precursor for *de novo* dTTP (thymidine triphosphate) synthesis. Thymidylate synthase converts dUMP to TMP and dTMP to dTDP. NDP (nucleoside diphosphate) kinase then converts dTDP into dTTP. This last reaction is among the second most highly upregulated reactions (see NDPK in Fig. 4A). Also upregulated is the production of deoxyguanosine, uracil, cytosine and adenosine triphosphates, which are the components of RNA. The nucleotide salvage pathway shows upregulated adenine recovery from RNA/DNA degradation by adenine phosphoribosyltransferase, which would coincide with an increased demand for adenine to bind to uracil. Within the pyrimidine synthesis pathway, three out of the top four upregulated pathways are involved in the production of dTTP. Proteomic‐informed flux data begin to show these same patterns at 72 hpi, apart from deoxyuridine kinase and thymidine kinase, which are upregulated at 48 hpi. Altogether, these results thus suggest increased viral RNA production.

### Altered energy metabolism for viral replication

For both sets of flux data, the components of the mitochondrial electron transport chain/oxidative phosphorylation are mostly downregulated through all the time points, indicating reduced ATP production from the citric acid cycle (TCA) [see transcriptomic‐informed results in (Fig. 4B) CI, CII, CIII, CIV, CV]. As shown in Fig. 3, the synthesis of ubiquinone, which is part of the mitochondrial electron transport chain, is progressively downregulated in the transcriptomic‐informed flux data and also downregulated in the proteomic‐informed flux data. Succinate dehydrogenase (SDH) is the only enzyme in the citric acid cycle that is also involved in the electron transport chain and is downregulated over all the time points in the transcriptomic‐informed flux data [75]. The resulting accumulation of succinate and fumarate led to an upsurge in mitochondrial transport of these metabolites into the cytosol (Fig. 3). Downregulation of the electron transport chain, including SDH, may indicate that COVID‐19 uses a similar mechanism to cancer cells to avoid oxidative stress initiating cell death while it is replicating [76, 77]. In the transcriptomic‐informed flux data, mitochondrial TCA reactions producing intermediates for amino acid synthesis are downregulated at 24 hpi, then become progressively upregulated for viral protein synthesis. Paradoxically, while citrate synthase is downregulated, the citrate shuttle and citrate lyase are upregulated providing acetyl‐CoA for fatty acid synthesis. The cytosolic reactions of the TCA are progressively upregulated through the time points, generating energy in the form of NADH/NADPH for fatty acid synthesis needed for the viral envelope. The NADPH producing enzymes in the pentose phosphate pathway, glucose 6‐phosphate dehydrogenase and phosphogluconate dehydrogenase, which also provide NADH/NADPH for fatty acid synthesis, are upregulated. These may have been influenced by maximising phosphogluconate dehydrogenase in the FVA. The proteomic‐informed flux data show very similar results for the TCA and pentose phosphate pathways.

In parallel, the majority of the reactions of glycolysis in both sets of flux data are upregulated or unchanged through all time points, with a dip in some upregulated reactions at 48 hpi. The main rate‐limiting enzyme of glycolysis, phosphofructokinase, is progressively upregulated through all time points in the transcriptomic‐informed flux data (see PFK in Fig. 4B). At the final 72‐h time point, the largest upregulation in the transcriptomic‐informed flux data occurs in the reaction catalysed by aldehyde dehydrogenase (see ALDD2 in Fig. 4B). Aldehyde dehydrogenase consumes the oxidative stressor acetaldehyde and produces energy in the form of NADH/NADPH. Interestingly, this reaction occurs in the cytosol, where viral replication takes place and is a known indicator of oxidative stress/lipid peroxidation [78]. The NADH from glycolysis is transported into the mitochondria to be used by the TCA. Transport is facilitated by both the malate‐aspartate shuttle, which is catalysed by the enzyme malate dehydrogenase, and the glycerol‐phosphate shuttle, which is catalysed by glycerol‐3‐phosphate dehydrogenase [79]. Here, in both sets of data, the flux of glycerol‐phosphate shuttle is unchanged, but the malate‐aspartate shuttle is upregulated, with the mitochondrial malate dehydrogenase reaction being the most highly upregulated in the transcriptomic‐informed TCA flux data. Within the glycolytic pathway for both sets of data, ATP production from pyruvate kinase is upregulated suggesting that glycolysis, rather than oxidative phosphorylation, is favoured by the virus for the generation of ATP for amino acid and RNA production [80].

Overall, proteomic‐informed flux data were in broad agreement with the transcriptomics‐informed flux data revealing greater influences on pathways at earlier time points. See Fig. 3B for a visual representation of the changes in the central energy metabolism.

### Dysregulation of fatty acid metabolism

The largest perturbation in fatty acid synthesis occurs in the downregulation of desaturation of fatty acids in the endoplasmic reticulum, where polyunsaturated fatty acids such as oleic acid are produced (Fig. 4D, RE3243R). This downregulation is significantly more pronounced in the transcriptomics‐informed flux data as compared to the proteomic‐informed flux data. The downregulation of unsaturated fatty acid synthesis is a known effect of enveloped virus infection [81]. Yan *et al*. [82] found that oleic acid was upregulated in coronavirus HCoV‐229E infection in HuH7 cells, though this study did not include COVID‐19. This result could either be a result of the missing phospholipase A2 pathway enzymes or indicate the downregulation of unsaturated fatty acids involved in the host cell immune response [83, 84].

Moreover, the elongation stage of fatty acid synthesis in the cytosol is upregulated at 72 hpi, which could be linked to the production of the viral cell envelope. In the transcriptomic‐informed flux data, there is indeed an upregulation 4 of the 5 steps of the synthesis of CoA (Fig. 3), which is needed for the citric acid cycle and fatty acid metabolism [85], in conjunction with a decrease in the activity of the majority of enzymes associated with β‐oxidation of fatty acids and cholesterol synthesis. The breakdown of γ‐linolenoyl‐CoA to octanoyl‐CoA is the only β‐oxidation reaction that shows an increase in activity in both sets of the flux data (Fig. 4D, FAOXC183806m). This reaction is the final step of β‐oxidation in the mitochondria and provides acetyl‐CoA for the citric acid cycle. In the transcriptomic‐informed flux data, the transfer of electrons from octanoyl‐CoA to ubiquinone is progressively downregulated showing further evidence of the downregulation of the electron transport chain (Fig. 4D, ETF/QO) [86]. These perturbations are more pronounced in the transcriptomics‐informed flux data as compared to the proteomic‐informed flux data.

### Secretome inflammatory biomarkers

As expected, protein secretion exhibited a widespread upregulation, though visible especially at 24 and 72 hpi, with 10 upregulated cytokine/chemokine secretory pathways. Macrophage inflammatory protein‐1β (MIP‐1β/CCL4), IP10/*CXCL10*, IL‐12B and eotaxin (*CCL11*) were found to be upregulated in the proteomic‐informed data, while in the transcriptomics‐informed data upregulation was found for IL‐6, IL‐8, IL‐12A, vascular endothelial growth factor A (VEGFA), chemokine (C‐C motif) ligand 20 (*CCL20*) and alpha‐1 acid glycoprotein (AGP). Analysis of the metabolic alterations further revealed high upregulation of IL‐17A, IL‐15 and IL‐7.

As shown in Fig. 4E, MIP‐1β was the most highly expressed cytokine/chemokine at 24 and 48 hpi and was then replaced by IP10 at 72 hpi. IP10 has been reported as a good biomarker of SARS‐CoV‐2 disease progression and severity [87, 88, 89] and has been implicated in the cytokine storm with higher levels leading to more severe disease [90, 91]. Eotaxin was the second most highly upregulated protein at 72 hpi followed by AGP and IL‐6, respectively. In the proteomic‐informed flux data, only eotaxin was upregulated at 72 hpi. MIP‐1β is a proinflammatory chemokine known to induce cytokines such as IL‐6 and TNF‐α and could be an early predictor of severe disease [92]. Eotaxin has the potential to act as a marker to predict disease severity and outcome. Although it is seen to progressively elevate in patients with mild symptoms, eosinopenia has been detected in patients with severe disease with a subsequent increase being associated with a good outcome [93, 94, 95]. AGP is an acute‐phase protein, and plasma levels of AGP have previously been found to be correlated with influenza disease progression and could be a potential novel biomarker for SARS‐CoV‐2 disease progression [96]. IL‐6 has been implicated in the cytokine storm and has received a lot of attention as a potential therapeutic target with clinical trials of IL‐6 receptor blockers tocilizumab, siltuximab and sarilumab currently underway [97, 98, 99, 100]. IL‐17A induces inflammatory cytokines involved in the cytokine storm such as IL‐6 and IL‐8 and has been linked with severe disease [101, 102]. IL‐17A blockers such as secukinumab have been proposed as a treatment for COVID‐19, although their administration would have to be timed carefully [103, 104]. IL‐7 has a role in maintaining NKT (natural killer T cells) and T cells in liver cells [105] and promotes cell survival [106]. IL‐15 is an important regulator of the immune response and a known antiviral cytokine [107, 108, 109]. Increased levels of IL‐15 may also be a biomarker of severe lymphopenia requiring longer hospital care [110]. The decrease in the production of hyaluronan shown in the pathway‐level flux average (Fig. 3A) is a further indicator of liver damage [111]. Interestingly, the increase in d‐alanine metabolism also detected in the metabolic fluxes may be a mechanism for producing cytotoxic oxidative stress as part of the innate immune response [112] (Fig. 3A).

### Significance for COVID‐19 treatments

Figure 4 shows a visual summary of the key metabolic changes identified at 72 hpi. Taken together, they can be utilised for hypothesising improved treatment strategies for COVID‐19 in cancer patients. The upregulated secretory proteins identified in this study provide some insights into therapeutic treatments for COVID‐19. Upregulation of IL‐7 is an indicator of T‐cell exhaustion and elevated inflammatory cytokine characteristics of COVID‐19 [113]. IL‐7 has a protective role in maintaining NKT (natural killer T cells) and T cells in liver cells [105] and promotes cell survival [106]. The levels of IL‐7 could be elevated in COVID‐19 patients by using IL7r (recombinant IL‐7) therapy, which has been used to treat HIV patients and was able to restore CD4+ T cells while being well‐tolerated [114]. Secondly, MitoQ (mitochondrial‐targeted ubiquinone) has powerful antioxidant effects that could be used as a treatment to protect against mitochondrial electron transport chain dysfunction/oxidative stress, compensating for the increased downregulation of ETC ubiquinone seen in COVID‐19 infection and restoring T‐cell homeostasis [115, 116]. Finally, our findings support dietary supplementation of unsaturated fatty acids administered orally or intravenously to counteract their downregulation by COVID‐19, which has been suggested as a treatment to suppress inflammation [83, 84, 117].

It should be noted that individuals suffering from cancer may already be taking medication. While the treatments suggested here do not interact with common liver cancer drugs according to the interactions listed in the Joint Formulary Committee [118] (Table S6), caution should be taken to ensure that any COVID‐19 treatments are compatible with existing treatment regimes.

## Conclusions

While transcriptomic‐only approaches can provide insights into changes in gene expression caused by viral infection, translating these data into metabolic data can give deeper and mechanistic insights into the effects of viral infection on the host cell. Here, we use genome‐scale‐metabolic modelling of Huh7 cells with transcriptomic‐ and proteomic‐informed FVA to explore the metabolic effects of altered gene expression from COVID‐19‐infected cancer cells on cellular phenotype. This method allows the identification of dysregulation at the enzyme/reaction level, in contrast to previous studies of metabolism. Our models were constrained to represent the viral‐infected cell using evidence from literature and the pathway analysis of DEGs.

A pathway analysis revealed an upregulation of PPARalpha, known to be involved in fatty acid metabolism and regulation of the immune system [58]. The analysis of the resulting transcriptomic‐ and proteomic‐informed flux data revealed perturbations in four main areas of metabolism, RNA synthesis, energy production, fatty acid synthesis and the secretome. Proteomic‐informed flux data sometimes displayed a cascade effect where patterns in reactions/pathways appeared at a later time point than with flux data. Energy production showed an increased shift from the TCA to glycolysis, as evidenced by the upregulation of key glycolytic enzymes and the downregulation of the mitochondrial electron transport chain. This pseudohypoxic metabolic shift is in addition to the dysregulation already seen in cancer cells and may contribute to the increased risk seen in cancer patients infected with COVID‐19. Reactions involved in RNA production were found to be upregulated in accordance with viral replication needs. While the production of polyunsaturated acids was downregulated, the synthesis of fatty acids needed for the viral envelope was upregulated. Key inflammatory secretory proteins involved in the cytokine storm were found to be upregulated including IP10/*CXCL10*, eotaxin, MIP‐1β and IL‐6. Finally, we suggest therapeutic treatments based on mediating the inflammatory response and metabolic key changes that enable the virus to replicate in the host cancer cell. Future studies could enhance our understanding by investigating multicellular metabolic changes as well as how individual data such as gender, age and ethnicity affect the metabolic changes caused by COVID‐19 infection. This will facilitate the investigation of more personalised therapeutic interventions for cancer patients.

## Author contributions

CA conceived and supervised the study; LB and GB performed the RNA‐seq data analyses; EY and GZ developed the software and performed the simulations; EY and GZ analysed the data; all authors contributed to drafting the manuscript; EY, GZ and CA produced the final version of the manuscript.

## Supporting information


**Table S1.** Differentially expressed genes and proteins.

**Table S2.** Enrichment analysis of differentially expressed genes.

**Table S3.** Enrichment analysis of differentially expressed proteins.

**Table S4.** Transcriptomic‐informed flux rates for reactions and pathways.

**Table S5.** Proteomic‐informed flux rates for reactions and pathways.

**Table S6.** Common liver cancer drugs and their interactions.

## Data Availability

All software and data produced in the study are available at: https://github.com/Angione‐Lab/Metabolic‐modelling‐of‐SARS‐CoV‐2‐in‐cancer‐cells.
