# Supplementary material for: Genome‐scale metabolic modelling of SARS‐CoV‐2 in cancer cells reveals an increased shift to glycolytic energy production
Source: FEBS Lett. 2021 Sep 5;595(18):2350–65. doi: 10.1002/1873-3468.14180 (PMC8427129; doi:10.1002/1873-3468.14180)
Supplement: Supplementary file 6 — Table S6. Common liver cancer drugs and their interactions. [file FEB2-595-2350-s003.pdf]

Supplementary Table 6: Common Liver Cancer Drugs and their Interactions

Drug	Interactions
Atezolizumab<br>Tecentriq (Atezolizumab)	Bacillus Calmette-Guérin vaccine<br>Betamethasone<br>Deflazacort<br>Dexamethasone<br>Hydrocortisone<br>Influenza vaccine (live)<br>Measles, mumps and rubella vaccine, live<br>Methylprednisolone<br>Prednisolone<br>Rotavirus vaccine<br>Typhoid vaccine, oral<br>Varicella-zoster vaccine<br>Yellow fever vaccine, live
Avastin (Bevacizumab)<br>Bevacizumab	Acalabrutinib<br>Aceclofenac<br>Acenocoumarol<br>Aldesleukin<br>Alteplase<br>Amsacrine<br>Anagrelide<br>Apixaban<br>Argatroban<br>Arsenic trioxide<br>Asparaginase<br>Aspirin<br>Axitinib<br>Azacitidine<br>Azathioprine<br>Bacillus Calmette-Guérin vaccine<br>Belatacept<br>Bendamustine<br>Bexarotene<br>Bivalirudin<br>Bleomycin<br>Bortezomib<br>Bosutinib<br>Bromfenac<br>Busulfan<br>Cabazitaxel<br>Cabozantinib<br>Cangrelor<br>Capecitabine<br>Caplacizumab<br>Carboplatin<br>Carfilzomib<br>Carmustine<br>Celecoxib<br>Ceritinib<br>Chlorambucil

	Cilostazol<br>Cisplatin<br>Citalopram<br>Cladribine<br>Clofarabine<br>Clopidogrel<br>Cobimetinib<br>Crisantaspase<br>Cyclophosphamide<br>Cytarabine<br>Dabigatran<br>Dacarbazine<br>Dactinomycin<br>Dalteparin<br>Danaparoid<br>Dapoxetine<br>Dasatinib<br>Daunorubicin<br>Decitabine<br>Dexketoprofen<br>Diclofenac<br>Dipyridamole<br>Docetaxel<br>Doxorubicin<br>Duloxetine<br>Edoxaban<br>Enoxaparin<br>Epirubicin<br>Epoprostenol<br>Eptifibatide<br>Eribulin<br>Escitalopram<br>Estramustine<br>Etodolac<br>Etoposide<br>Etoricoxib<br>Fludarabine<br>Fluorouracil<br>Fluoxetine<br>Flurbiprofen<br>Fluvoxamine<br>Fondaparinux<br>Ganciclovir<br>Gemcitabine<br>Gemtuzumab ozogamicin<br>Heparin<br>Hydroxycarbamide<br>Ibrutinib<br>Ibuprofen<br>Idarubicin<br>Ifosfamide

	Iloprost<br>Imatinib<br>Indometacin<br>Influenza vaccine (live)<br>Inotersen<br>Irinotecan<br>Ketoprofen<br>Ketorolac<br>Leflunomide<br>Lenalidomide<br>Lenvatinib<br>Lomustine<br>Measles, mumps and rubella vaccine, live<br>Mefenamic acid<br>Meloxicam<br>Melphalan<br>Mercaptopurine<br>Methotrexate<br>Mifamurtide<br>Mitomycin<br>Mitotane<br>Mitoxantrone<br>Nabumetone<br>Naproxen<br>Nelarabine<br>Nicotinic acid<br>Nilotinib<br>Nintedanib<br>Niraparib<br>Olaparib<br>Omega-3-acid ethyl esters<br>Oxaliplatin<br>Paclitaxel<br>Palbociclib<br>Panobinostat<br>Parecoxib<br>Paroxetine<br>Pegaspargase<br>Peginterferon alfa<br>Pemetrexed<br>Pentostatin<br>Phenazone<br>Phenindione<br>Piroxicam<br>Pixantrone<br>Pomalidomide<br>Ponatinib<br>Prasugrel<br>Procarbazine<br>Raltitrexed<br>Regorafenib

	Ribociclib<br>Rivaroxaban<br>Rotavirus vaccine<br>Rucaparib<br>Ruxolitinib<br>Sertraline<br>Sorafenib<br>Streptokinase<br>Streptozocin<br>Sulfasalazine<br>Sulindac<br>Sunitinib<br>Talazoparib<br>Tegafur<br>Temozolomide<br>Temsirolimus<br>Tenecteplase<br>Tenoxicam<br>Thalidomide<br>Thiotepa<br>Tiaprofenic acid<br>Ticagrelor<br>Tinzaparin<br>Tioguanine<br>Tirofiban<br>Tolfenamic acid<br>Topotecan<br>Trabectedin<br>Trametinib<br>Treosulfan<br>Treprostinil<br>Typhoid vaccine, oral<br>Urokinase<br>Valganciclovir<br>Vandetanib<br>Varicella-zoster vaccine<br>Venlafaxine<br>Vinblastine<br>Vincristine<br>Vindesine<br>Vinflunine<br>Vinorelbine<br>Volanesorsen<br>Vortioxetine<br>Warfarin<br>Yellow fever vaccine, live
Cabometyx (Cabozantinib-S-Malate)<br>Cabozantinib-S-Malate	Acalabrutinib<br>Aceclofenac<br>Acenocoumarol

	Adalimumab<br>Aldesleukin<br>Alemtuzumab<br>Alteplase<br>Amifampridine<br>Aminophylline<br>Amiodarone<br>Amisulpride<br>Amphotericin B<br>Amsacrine<br>Anagrelide<br>Apalutamide<br>Apixaban<br>Apomorphine<br>Aprepitant<br>Argatroban<br>Arsenic trioxide<br>Artemether<br>Artenimol<br>Asparaginase<br>Aspirin<br>Atazanavir<br>Axitinib<br>Azacitidine<br>Azathioprine<br>Bambuterol<br>Beclometasone<br>Bedaquiline<br>Belatacept<br>Bendamustine<br>Bendroflumethiazide<br>Betamethasone<br>Bevacizumab<br>Bexarotene<br>Bivalirudin<br>Bleomycin<br>Blinatumomab<br>Bortezomib<br>Bosentan<br>Bosutinib<br>Brentuximab vedotin<br>Bromfenac<br>Budesonide<br>Bumetanide<br>Busulfan<br>Cabazitaxel<br>Canakinumab<br>Cangrelor<br>Capecitabine<br>Caplacizumab<br>Carbamazepine

	Carboplatin<br>Carfilzomib<br>Carmustine<br>Celecoxib<br>Ceritinib<br>Certolizumab pegol<br>Chlorambucil<br>Chlorothiazide<br>Chlorpromazine<br>Chlortalidone<br>Cilostazol<br>Cisplatin<br>Citalopram<br>Cladribine<br>Clarithromycin<br>Clofarabine<br>Clomipramine<br>Clopidogrel<br>Cobicistat<br>Cobimetinib<br>Crisantaspase<br>Crizotinib<br>Cyclophosphamide<br>Cytarabine<br>Dabigatran<br>Dabrafenib<br>Dacarbazine<br>Dactinomycin<br>Dalteparin<br>Danaparoid<br>Dapoxetine<br>Daratumumab<br>Darunavir<br>Dasatinib<br>Daunorubicin<br>Decitabine<br>Deflazacort<br>Delamanid<br>Desflurane<br>Dexamethasone<br>Dexketoprofen<br>Dexrazoxane<br>Diclofenac<br>Diltiazem<br>Dinutuximab<br>Dipyridamole<br>Disopyramide<br>Docetaxel<br>Domperidone<br>Doxorubicin<br>Dronedarone

	Droperidol<br>Duloxetine<br>Edoxaban<br>Efavirenz<br>Encorafenib<br>Enoxaparin<br>Entrectinib<br>Enzalutamide<br>Epirubicin<br>Epoprostenol<br>Eptifibatide<br>Eribulin<br>Erythromycin<br>Escitalopram<br>Estramustine<br>Etodolac<br>Etoposide<br>Etoricoxib<br>Fingolimod<br>Flecainide<br>Fluconazole<br>Fludarabine<br>Fludrocortisone<br>Fluorouracil<br>Fluoxetine<br>Flurbiprofen<br>Fluvoxamine<br>Fondaparinux<br>Formoterol<br>Fosamprenavir<br>Fosphenytoin<br>Furosemide<br>Ganciclovir<br>Gemcitabine<br>Gemtuzumab ozogamicin<br>Glasdegib<br>Golimumab<br>Granisetron<br>Grapefruit<br>Haloperidol<br>Heparin<br>Hydrochlorothiazide<br>Hydrocortisone<br>Hydroflumethiazide<br>Hydroxycarbamide<br>Hydroxyzine<br>Ibrutinib<br>Ibuprofen<br>Idarubicin<br>Idelalisib<br>Ifosfamide

	Iloprost<br>Imatinib<br>Indacaterol<br>Indapamide<br>Indometacin<br>Infliximab<br>Inotersen<br>Inotuzumab ozogamicin<br>Ipilimumab<br>Irinotecan<br>Isavuconazole<br>Isoflurane<br>Itraconazole<br>Ivabradine<br>Ketoconazole<br>Ketoprofen<br>Ketorolac<br>Lapatinib<br>Leflunomide<br>Lenalidomide<br>Lenvatinib<br>Letermovir<br>Levomepromazine<br>Lithium<br>Lofexidine<br>Lomustine<br>Lopinavir<br>Mefenamic acid<br>Mefloquine<br>Meloxicam<br>Melphalan<br>Mercaptopurine<br>Methadone<br>Methotrexate<br>Methylprednisolone<br>Metolazone<br>Midazolam<br>Mitomycin<br>Mitotane<br>Mitoxantrone<br>Mizolastine<br>Mogamulizumab<br>Moxifloxacin<br>Nabumetone<br>Naproxen<br>Nelarabine<br>Netupitant<br>Nevirapine<br>Nicotinic acid<br>Nilotinib<br>Nintedanib

	Niraparib<br>Nivolumab<br>Obinutuzumab<br>Olaparib<br>Olodaterol<br>Omega-3-acid ethyl esters<br>Ondansetron<br>Osimertinib<br>Oxaliplatin<br>Ozanimod<br>Paclitaxel<br>Palbociclib<br>Paliperidone<br>Palonosetron<br>Panobinostat<br>Parecoxib<br>Paroxetine<br>Pasireotide<br>Pazopanib<br>Pegaspargase<br>Peginterferon alfa<br>Pembrolizumab<br>Pemetrexed<br>Pentamidine<br>Pentostatin<br>Phenazone<br>Phenindione<br>Phenobarbital<br>Phenytoin<br>Pimozide<br>Piroxicam<br>Pixantrone<br>Pomalidomide<br>Ponatinib<br>Posaconazole<br>Prasugrel<br>Prednisolone<br>Primidone<br>Procarbazine<br>Quinine<br>Raltitrexed<br>Ramucirumab<br>Ranolazine<br>Regorafenib<br>Ribociclib<br>Rifampicin<br>Risperidone<br>Ritonavir<br>Rituximab<br>Rivaroxaban<br>Rucaparib

	Ruxolitinib<br>Salbutamol<br>Salmeterol<br>Sertraline<br>Sevoflurane<br>Sildenafil<br>Siponimod<br>Sorafenib<br>Sotalol<br>St John's wort<br>Streptokinase<br>Streptozocin<br>Sulfasalazine<br>Sulindac<br>Sulpiride<br>Sunitinib<br>Talazoparib<br>Tegafur<br>Telavancin<br>Temozolomide<br>Temsirolimus<br>Tenecteplase<br>Tenoxicam<br>Terbutaline<br>Tetrabenazine<br>Thalidomide<br>Theophylline<br>Thiotepa<br>Tiaprofenic acid<br>Ticagrelor<br>Tinzaparin<br>Tioguanine<br>Tipranavir<br>Tirofiban<br>Tizanidine<br>Tolfenamic acid<br>Tolterodine<br>Topotecan<br>Torasemide<br>Toremifene<br>Trabectedin<br>Trametinib<br>Trastuzumab<br>Trastuzumab deruxtecan<br>Trastuzumab emtansine<br>Treosulfan<br>Treprostinil<br>Triamcinolone<br>Urokinase<br>Valganciclovir<br>Vandetanib

	Vardenafil<br>Vemurafenib<br>Venlafaxine<br>Verapamil<br>Vernakalant<br>Vilanterol<br>Vinblastine<br>Vincristine<br>Vindesine<br>Vinflunine<br>Vinorelbine<br>Volanesorsen<br>Voriconazole<br>Vortioxetine<br>Warfarin<br>Xipamide<br>Zuclopenthixol
Cyramza (Ramucirumab)<br>Ramucirumab	Aldesleukin<br>Amsacrine<br>Arsenic trioxide<br>Asparaginase<br>Axitinib<br>Azacitidine<br>Azathioprine<br>Bacillus Calmette-Guérin vaccine<br>Belatacept<br>Bendamustine<br>Bexarotene<br>Bleomycin<br>Bortezomib<br>Bosutinib<br>Busulfan<br>Cabazitaxel<br>Cabozantinib<br>Capecitabine<br>Carboplatin<br>Carfilzomib<br>Carmustine<br>Ceritinib<br>Chlorambucil<br>Cisplatin<br>Cladribine<br>Clofarabine<br>Crisantaspase<br>Cyclophosphamide<br>Cytarabine<br>Dacarbazine<br>Dactinomycin<br>Dasatinib<br>Daunorubicin

	Decitabine<br>Dexrazoxane<br>Docetaxel<br>Doxorubicin<br>Epirubicin<br>Eribulin<br>Estramustine<br>Etoposide<br>Fludarabine<br>Fluorouracil<br>Ganciclovir<br>Gemcitabine<br>Gemtuzumab ozogamicin<br>Hydroxycarbamide<br>Ibrutinib<br>Idarubicin<br>Ifosfamide<br>Imatinib<br>Influenza vaccine (live)<br>Irinotecan<br>Leflunomide<br>Lenalidomide<br>Lomustine<br>Measles, mumps and rubella vaccine, live<br>Melphalan<br>Mercaptopurine<br>Methotrexate<br>Mifamurtide<br>Mitomycin<br>Mitotane<br>Mitoxantrone<br>Nelarabine<br>Nilotinib<br>Niraparib<br>Olaparib<br>Oxaliplatin<br>Paclitaxel<br>Palbociclib<br>Panobinostat<br>Pegaspargase<br>Peginterferon alfa<br>Pemetrexed<br>Pentostatin<br>Pixantrone<br>Pomalidomide<br>Procarbazine<br>Raltitrexed<br>Regorafenib<br>Ribociclib<br>Rotavirus vaccine<br>Rucaparib

	Ruxolitinib<br>Sorafenib<br>Streptozocin<br>Sulfasalazine<br>Sunitinib<br>Talazoparib<br>Tegafur<br>Temozolomide<br>Temsirolimus<br>Thalidomide<br>Thiotepa<br>Tioguanine<br>Topotecan<br>Trabectedin<br>Treosulfan<br>Typhoid vaccine, oral<br>Valganciclovir<br>Varicella-zoster vaccine<br>Vinblastine<br>Vincristine<br>Vindesine<br>Vinflunine<br>Vinorelbine<br>Yellow fever vaccine, live
Keytruda (Pembrolizumab)<br>Pembrolizumab	Aldesleukin<br>Amsacrine<br>Arsenic trioxide<br>Asparaginase<br>Axitinib<br>Azacitidine<br>Azathioprine<br>Bacillus Calmette-Guérin vaccine<br>Belatacept<br>Bendamustine<br>Betamethasone<br>Bexarotene<br>Bleomycin<br>Bortezomib<br>Bosutinib<br>Busulfan<br>Cabazitaxel<br>Cabozantinib<br>Capecitabine<br>Carboplatin<br>Carfilzomib<br>Carmustine<br>Ceritinib<br>Chlorambucil<br>Cisplatin<br>Cladribine

	Clofarabine<br>Crisantaspase<br>Cyclophosphamid<br>Cytarabine<br>Dacarbazine<br>Dactinomycin<br>Dasatinib<br>Daunorubicin<br>Decitabine<br>Deflazacort<br>Dexamethasone<br>Dexrazoxane<br>Docetaxel<br>Doxorubicin<br>Epirubicin<br>Eribulin<br>Estramustine<br>Etoposide<br>Fludarabine<br>Fluorouracil<br>Ganciclovir<br>Gemcitabine<br>Gemtuzumab ozogamicin<br>Hydrocortisone<br>Hydroxycarbamide<br>Ibrutinib<br>Idarubicin<br>Ifosfamide<br>Imatinib<br>Influenza vaccine (live)<br>Irinotecan<br>Leflunomide<br>Lenalidomide<br>Lomustine<br>Measles, mumps and rubella vaccine, live<br>Melphalan<br>Mercaptopurine<br>Methotrexate<br>Methylprednisolone<br>Mifamurtide<br>Mitomycin<br>Mitotane<br>Mitoxantrone<br>Nelarabine<br>Nilotinib<br>Niraparib<br>Olaparib<br>Oxaliplatin<br>Paclitaxel<br>Palbociclib<br>Panobinostat

	Pegaspargase<br>Peginterferon alfa<br>Pemetrexed<br>Pentostatin<br>Pixantrone<br>Pomalidomide<br>Prednisolone<br>Procarbazine<br>Raltitrexed<br>Regorafenib<br>Ribociclib<br>Rotavirus vaccine<br>Rucaparib<br>Ruxolitinib<br>Sorafenib<br>Streptozocin<br>Sulfasalazine<br>Sunitinib<br>Talazoparib<br>Tegafur<br>Temozolomide<br>Temsirolimus<br>Thalidomide<br>Thiotepa<br>Tioguanine<br>Topotecan<br>Trabectedin<br>Treosulfan<br>Typhoid vaccine, oral<br>Valganciclovir<br>Varicella-zoster vaccine<br>Vinblastine<br>Vincristine<br>Vindesine<br>Vinflunine<br>Vinorelbine<br>Yellow fever vaccine, live
Lenvatinib Mesylate<br>Lenvima (Lenvatinib Mesylate)	Acalabrutinib<br>Aceclofenac<br>Acenocoumarol<br>Alteplase<br>Amifampridine<br>Aminophylline<br>Amiodarone<br>Amisulpride<br>AmphotericinB<br>Anagrelide<br>Apalutamide<br>Apixaban<br>Apomorphine<br>Argatroban

	Arsenictrioxide<br>Artemether<br>Artenimol<br>Aspirin<br>Axitinib<br>Bambuterol<br>Beclometasone<br>Bedaquiline<br>Bendroflumethiazide<br>Betamethasone<br>Bevacizumab<br>Bivalirudin<br>Bosutinib<br>Bromfenac<br>Budesonide<br>Bumetanide<br>Cabozantinib<br>Cangrelor<br>Caplacizumab<br>Celecoxib<br>Ceritinib<br>Chlorothiazide<br>Chlorpromazine<br>Chlortalidone<br>Cilostazol<br>Citalopram<br>Clarithromycin<br>Clomipramine<br>Clopidogrel<br>Cobimetinib<br>Crizotinib<br>Dabigatran<br>Dalteparin<br>Danaparoid<br>Dapoxetine<br>Dasatinib<br>Deflazacort<br>Delamanid<br>Desflurane<br>Dexamethasone<br>Dexketoprofen<br>Diclofenac<br>Dipyridamole<br>Disopyramide<br>Domperidone<br>Dronedarone<br>Droperidol<br>Duloxetine<br>Edoxaban<br>Efavirenz<br>Encorafenib

	Enoxaparin<br>Entrectinib<br>Epoprostenol<br>Eptifibatide<br>Eribulin<br>Erythromycin<br>Escitalopram<br>Etodolac<br>Etoricoxib<br>Fingolimod<br>Flecainide<br>Fluconazole<br>Fludrocortisone<br>Fluoxetine<br>Flurbiprofen<br>Fluvoxamine<br>Fondaparinux<br>Formoterol<br>Furosemide<br>Glasdegib<br>Granisetron<br>Haloperidol<br>Heparin<br>Hydrochlorothiazide<br>Hydrocortisone<br>Hydroflumethiazide<br>Hydroxyzine<br>Ibrutinib<br>Ibuprofen<br>Iloprost<br>Indacaterol<br>Indapamide<br>Indometacin<br>Inotersen<br>Inotuzumabozogamicin<br>Isoflurane<br>Ivabradine<br>Ketoprofen<br>Ketorolac<br>Lapatinib<br>Levomepromazine<br>Lithium<br>Lofexidine<br>Mefenamicacid<br>Mefloquine<br>Meloxicam<br>Methadone<br>Methylprednisolone<br>Metolazone<br>Mizolastine<br>Moxifloxacin

	Nabumetone<br>Naproxen<br>Nicotinicacid<br>Nilotinib<br>Nintedanib<br>Olodaterol<br>Omega--acidethylesters<br>Ondansetron<br>Osimertinib<br>Ozanimod<br>Paliperidone<br>Palonosetron<br>Panobinostat<br>Parecoxib<br>Paroxetine<br>Pasireotide<br>Pazopanib<br>Pentamidine<br>Phenazone<br>Phenindione<br>Pimozide<br>Piroxicam<br>Ponatinib<br>Prasugrel<br>Prednisolone<br>Quinine<br>Ranolazine<br>Regorafenib<br>Ribociclib<br>Risperidone<br>Rivaroxaban<br>Ruxolitinib<br>Salbutamol<br>Salmeterol<br>Sertraline<br>Sevoflurane<br>Sildenafil<br>Siponimod<br>Sorafenib<br>Sotalol<br>Streptokinase<br>Sulindac<br>Sulpiride<br>Sunitinib<br>Telavancin<br>Tenecteplase<br>Tenoxicam<br>Terbutaline<br>Tetrabenazine<br>Theophylline<br>Tiaprofenicacid

	Ticagrelor<br>Tinzaparin<br>Tirofiban<br>Tizanidine<br>Tolfenamicacid<br>Tolterodine<br>Torasemide<br>Toremifene<br>Trametinib<br>Trastuzumabemtansine<br>Treprostinil<br>Triamcinolone<br>Urokinase<br>Vandetanib<br>Vardenafil<br>Vemurafenib<br>Venlafaxine<br>Vernakalant<br>Vilanterol<br>Vinflunine<br>Volanesorsen<br>Voriconazole<br>Vortioxetine<br>Warfarin<br>Xipamide<br>Zuclopenthixol
Nexavar (Sorafenib Tosylate)<br>Sorafenib Tosylate	Acenocoumarol<br>Adalimumab<br>Aldesleukin<br>Alemtuzumab<br>Amifampridine<br>Aminophylline<br>Amiodarone<br>Amisulpride<br>Amphotericin B<br>Amsacrine<br>Anagrelide<br>Apalutamide<br>Apomorphine<br>Arsenic trioxide<br>Artemether<br>Artenimol<br>Asparaginase<br>Axitinib<br>Azacitidine<br>Azathioprine<br>Bambuterol<br>Beclometasone<br>Bedaquiline<br>Belatacept<br>Bendamustine

	Bendroflumethiazide<br>Betamethasone<br>Bevacizumab<br>Bexarotene<br>Bleomycin<br>Blinatumomab<br>Bortezomib<br>Bosentan<br>Bosutinib<br>Brentuximab vedotin<br>Budesonide<br>Bumetanide<br>Busulfan<br>Cabazitaxel<br>Cabozantinib<br>Canakinumab<br>Capecitabine<br>Carbamazepine<br>Carboplatin<br>Carfilzomib<br>Carmustine<br>Ceritinib<br>Certolizumab pegol<br>Chlorambucil<br>Chlorothiazide<br>Chlorpromazine<br>Chlortalidone<br>Cisplatin<br>Citalopram<br>Cladribine<br>Clarithromycin<br>Clofarabine<br>Clomipramine<br>Crisantaspase<br>Crizotinib<br>Cyclophosphamide<br>Cytarabine<br>Dabrafenib<br>Dacarbazine<br>Dactinomycin<br>Daratumumab<br>Dasatinib<br>Daunorubicin<br>Decitabine<br>Deflazacort<br>Delamanid<br>Desflurane<br>Dexamethasone<br>Dexrazoxane<br>Dinutuximab<br>Disopyramide

	Docetaxel<br>Domperidone<br>Doxorubicin<br>Dronedarone<br>Droperidol<br>Efavirenz<br>Encorafenib<br>Entrectinib<br>Enzalutamide<br>Epirubicin<br>Eribulin<br>Erythromycin<br>Escitalopram<br>Eslicarbazepine<br>Estramustine<br>Etoposide<br>Fingolimod<br>Flecainide<br>Fluconazole<br>Fludarabine<br>Fludrocortisone<br>Fluorouracil<br>Formoterol<br>Fosphenytoin<br>Furosemide<br>Ganciclovir<br>Gemcitabine<br>Gemtuzumab ozogamicin<br>Glasdegib<br>Golimumab<br>Granisetron<br>Haloperidol<br>Hydrochlorothiazide<br>Hydrocortisone<br>Hydroflumethiazide<br>Hydroxycarbamide<br>Hydroxyzine<br>Ibrutinib<br>Idarubicin<br>Ifosfamide<br>Imatinib<br>Indacaterol<br>Indapamide<br>Infliximab<br>Inotuzumab ozogamicin<br>Ipilimumab<br>Irinotecan<br>Isoflurane<br>Ivabradine<br>Lapatinib<br>Leflunomide

	Lenalidomide<br>Lenvatinib<br>Levomepromazine<br>Lithium<br>Lofexidine<br>Lomustine<br>Mefloquine<br>Melphalan<br>Mercaptopurine<br>Methadone<br>Methotrexate<br>Methylprednisolone<br>Metolazone<br>Mifamurtide<br>Mitomycin<br>Mitotane<br>Mitoxantrone<br>Mizolastine<br>Mogamulizumab<br>Moxifloxacin<br>Nelarabine<br>Neomycin<br>Nevirapine<br>Nilotinib<br>Niraparib<br>Nivolumab<br>Obinutuzumab<br>Olaparib<br>Olodaterol<br>Ondansetron<br>Osimertinib<br>Oxaliplatin<br>Oxcarbazepine<br>Ozanimod<br>Paclitaxel<br>Palbociclib<br>Paliperidone<br>Palonosetron<br>Panobinostat<br>Pasireotide<br>Pazopanib<br>Pegaspargase<br>Peginterferon alfa<br>Pembrolizumab<br>Pemetrexed<br>Pentamidine<br>Pentostatin<br>Phenindione<br>Phenobarbital<br>Phenytoin<br>Pimozide

	Pixantrone<br>Pomalidomide<br>Prednisolone<br>Primidone<br>Procarbazine<br>Quinine<br>Raltitrexed<br>Ramucirumab<br>Ranolazine<br>Regorafenib<br>Ribociclib<br>Rifampicin<br>Risperidone<br>Rituximab<br>Rucaparib<br>Ruxolitinib<br>Salbutamol<br>Salmeterol<br>Sevoflurane<br>Sildenafil<br>Siponimod<br>Sotalol<br>St John's wort<br>Streptozocin<br>Sulfasalazine<br>Sulpiride<br>Sunitinib<br>Talazoparib<br>Tegafur<br>Telavancin<br>Temozolomide<br>Temsirolimus<br>Terbutaline<br>Tetrabenazine<br>Thalidomide<br>Theophylline<br>Thiotepa<br>Tioguanine<br>Tizanidine<br>Tolterodine<br>Topotecan<br>Torasemide<br>Toremifene<br>Trabectedin<br>Trastuzumab<br>Trastuzumab deruxtecan<br>Trastuzumab emtansine<br>Treosulfan<br>Triamcinolone<br>Valganciclovir<br>Vandetanib

	Vardenafil<br>Vemurafenib<br>Venlafaxine<br>Vernakalant<br>Vilanterol<br>Vinblastine<br>Vincristine<br>Vindesine<br>Vinflunine<br>Vinorelbine<br>Voriconazole<br>Warfarin<br>Xipamide<br>Zuclopenthixol
Nivolumab<br>Opdivo (Nivolumab)	Aldesleukin<br>Amsacrine<br>Arsenic trioxide<br>Asparaginase<br>Axitinib<br>Azacitidine<br>Azathioprine<br>Bacillus Calmette-Guérin vaccine<br>Belatacept<br>Bendamustine<br>Betamethasone<br>Bexarotene<br>Bleomycin<br>Bortezomib<br>Bosutinib<br>Busulfan<br>Cabazitaxel<br>Cabozantinib<br>Capecitabine<br>Carboplatin<br>Carfilzomib<br>Carmustine<br>Ceritinib<br>Chlorambucil<br>Cisplatin<br>Cladribine<br>Clofarabine<br>Crisantaspase<br>Cyclophosphamide<br>Cytarabine<br>Dacarbazine<br>Dactinomycin<br>Dasatinib<br>Daunorubicin<br>Decitabine<br>Deflazacort<br>Dexamethasone

	Dexrazoxane<br>Docetaxel<br>Doxorubicin<br>Epirubicin<br>Eribulin<br>Estramustine<br>Etoposide<br>Fludarabine<br>Fluorouracil<br>Ganciclovir<br>Gemcitabine<br>Gemtuzumab ozogamicin<br>Hydrocortisone<br>Hydroxycarbamide<br>Ibrutinib<br>Idarubicin<br>Ifosfamide<br>Imatinib<br>Influenza vaccine (live)<br>Irinotecan<br>Leflunomide<br>Lenalidomide<br>Lomustine<br>Measles, mumps and rubella vaccine, live<br>Melphalan<br>Mercaptopurine<br>Methotrexate<br>Methylprednisolone<br>Mifamurtide<br>Mitomycin<br>Mitotane<br>Mitoxantrone<br>Nelarabine<br>Nilotinib<br>Niraparib<br>Olaparib<br>Oxaliplatin<br>Paclitaxel<br>Palbociclib<br>Panobinostat<br>Pegaspargase<br>Peginterferon alfa<br>Pemetrexed<br>Pentostatin<br>Pixantrone<br>Pomalidomide<br>Prednisolone<br>Procarbazine<br>Raltitrexed<br>Regorafenib<br>Ribociclib

	Rotavirus vaccine<br>Rucaparib<br>Ruxolitinib<br>Sorafenib<br>Streptozocin<br>Sulfasalazine<br>Sunitinib<br>Talazoparib<br>Tegafur<br>Temozolomide<br>Temsirolimus<br>Thalidomide<br>Thiotepa<br>Tioguanine<br>Topotecan<br>Trabectedin<br>Treosulfan<br>Typhoid vaccine, oral<br>Valganciclovir<br>Varicella-zoster vaccine<br>Vinblastine<br>Vincristine<br>Vindesine<br>Vinflunine<br>Vinorelbine<br>Yellow fever vaccine, live
Regorafenib<br>Stivarga (Regorafenib)	Acalabrutinib<br>Aceclofenac<br>Acenocoumarol<br>Adalimumab<br>Aldesleukin<br>Alemtuzumab<br>Alteplase<br>Amsacrine<br>Anagrelide<br>Apalutamide<br>Apixaban<br>Aprepitant<br>Argatroban<br>Arsenictrioxide<br>Asparaginase<br>Aspirin<br>Atazanavir<br>Atorvastatin<br>Axitinib<br>Azacitidine<br>Azathioprine<br>Belatacept<br>Bendamustine<br>Bevacizumab<br>Bexarotene

	Bivalirudin<br>Bleomycin<br>Blinatumomab<br>Bortezomib<br>Bosentan<br>Bosutinib<br>Brentuximabvedotin<br>Bromfenac<br>Busulfan<br>Cabazitaxel<br>Cabozantinib<br>Canakinumab<br>Cangrelor<br>Capecitabine<br>Caplacizumab<br>Carbamazepine<br>Carboplatin<br>Carfilzomib<br>Carmustine<br><br><br>Celecoxib<br><br><br>Ceritinib<br>Certolizumabpegol<br>Chlorambucil<br>Cilostazol<br>Cisplatin<br>Citalopram<br>Cladribine<br>Clarithromycin<br>Clofarabine<br>Clopidogrel<br>Cobicistat<br>Cobimetinib<br>Crisantaspase<br>Crizotinib<br>Cyclophosphamide<br>Cytarabine<br>Dabigatran<br>Dabrafenib<br>Dacarbazine<br>Dactinomycin<br>Dalteparin<br>Danaparoid<br>Dapoxetine<br>Daratumumab<br>Darunavir<br>Dasatinib<br>Daunorubicin

	Decitabine<br>Dexketoprofen<br>Dexrazoxane<br>Diclofenac<br>Diltiazem<br>Dinutuximab<br>Dipyridamole<br>Docetaxel<br>Doxorubicin<br>Dronedarone<br>Duloxetine<br>Edoxaban<br>Efavirenz<br>Enoxaparin<br>Enzalutamide<br>Epirubicin<br>Epoprostenol<br>Eptifibatide<br>Eribulin<br>Erythromycin<br>Escitalopram<br>Estramustine<br>Etodolac<br>Etoposide<br>Etoricoxib<br>Fluconazole<br>Fludarabine<br>Fluorouracil<br>Fluoxetine<br>Flurbiprofen<br>Fluvastatin<br>Fluvoxamine<br>Fondaparinux<br>Fosamprenavir<br>Fosphenytoin<br>Ganciclovir<br>Gemcitabine<br>Gemtuzumabozogamicin<br>Golimumab<br>Grapefruit<br>Heparin<br>Hydroxycarbamide<br>Ibrutinib<br>Ibuprofen<br>Idarubicin<br>Idelalisib<br>Ifosfamide<br>Iloprost<br>Imatinib<br>Indometacin<br>Infliximab

	Inotersen<br>Inotuzumabozogamicin<br>Ipilimumab<br>Irinotecan<br>Isavuconazole<br>Itraconazole<br>Ketoconazole<br>Ketoprofen<br>Ketorolac<br>Leflunomide<br>Lenalidomide<br>Lenvatinib<br>Letermovir<br>Lomustine<br>Lopinavir<br>Mefenamicacid<br>Meloxicam<br>Melphalan<br>Mercaptopurine<br>Methotrexate<br>Mifamurtide<br>Mitomycin<br>Mitotane<br>Mitoxantrone<br>Mogamulizumab<br>Nabumetone<br>Naproxen<br>Nelarabine<br>Netupitant<br>Nevirapine<br>Nicotinicacid<br>Nilotinib<br>Nintedanib<br>Niraparib<br>Nivolumab<br>Obinutuzumab<br>Olaparib<br>Omega--acidethylesters<br>Oxaliplatin<br>Paclitaxel<br>Palbociclib<br>Panobinostat<br>Parecoxib<br>Paroxetine<br>Pegaspargase<br>Peginterferonalfa<br>Pembrolizumab<br>Pemetrexed<br>Pentostatin<br>Phenazone<br>Phenindione

	Phenobarbital<br>Phenytoin<br>Piroxicam<br>Pixantrone<br>Pomalidomide<br>Ponatinib<br>Posaconazole<br>Prasugrel<br>Primidone<br>Procarbazine<br>Raltitrexed<br>Ramucirumab<br>Ribociclib<br>Rifampicin<br>Ritonavir<br>Rituximab<br>Rivaroxaban<br>Rosuvastatin<br>Rucaparib<br>Ruxolitinib<br>Sertraline<br>Sorafenib<br>StJohn'swort<br>Streptokinase<br>Streptozocin<br>Sulfasalazine<br>Sulindac<br>Sunitinib<br>Talazoparib<br>Tegafur<br>Temozolomide<br>Temsirolimus<br>Tenecteplase<br>Tenoxicam<br>Thalidomide<br>Thiotepa<br>Tiaprofenicacid<br>Ticagrelor<br>Tinzaparin<br>Tioguanine<br>Tipranavir<br>Tirofiban<br>Tolfenamicacid<br>Topotecan<br>Trabectedin<br>Trametinib<br>Trastuzumab<br>Trastuzumabderuxtecan<br>Trastuzumabemtansine<br>Treosulfan<br>Treprostinil

	Urokinase<br>Valganciclovir<br>Vandetanib<br>Venlafaxine<br>Verapamil<br>Vinblasti<br>Vincristine<br>Vindesine<br>Vinflunine<br>Vinorelbine<br>Volanesorsen<br>Voriconazole<br>Vortioxetine<br>Warfarin
